# Characterization of Bacteria in Ballast Water Using MALDI-TOF Mass Spectrometry

**DOI:** 10.1371/journal.pone.0038515

**Published:** 2012-06-07

**Authors:** Kaveh Emami, Vahid Askari, Matthias Ullrich, Khwajah Mohinudeen, Arga Chandrashekar Anil, Lidita Khandeparker, J. Grant Burgess, Ehsan Mesbahi

**Affiliations:** 1 School of Biology, Newcastle University, Newcastle upon Tyne, United Kingdom; 2 School of Science and Engineering, Teesside University, Middlesbrough, Tees Valley, United Kingdom; 3 Molecular Life Science Research Center, Jacobs University Bremen, Bremen, Germany; 4 NEPAF Proteome Analysis Facility, Newcastle University, Newcastle upon Tyne, United Kingdom; 5 National Institute of Oceanography, Dona Paula, India; 6 School of Marine Science and Technology, Newcastle University, Newcastle upon Tyne, United Kingdom; J. Craig Venter Institute, United States of America

## Abstract

To evaluate a rapid and cost-effective method for monitoring bacteria in ballast water, several marine bacterial isolates were characterized by matrix-assisted laser desorption ionization-time of flight mass spectrometry (MALDI-TOF MS). Since International Maritime Organization (IMO) regulations are concerned with the unintended transportation of pathogenic bacteria through ballast water, emphasis was placed on detecting species of *Vibrio,* enterococci and coliforms. Seawater samples collected from the North Sea were incubated in steel ballast tanks and the presence of potentially harmful species of *Pseudomonas* was also investigated. At the genus-level, the identification of thirty six isolates using MALDI-TOF MS produced similar results to those obtained by 16S rRNA gene sequencing. No pathogenic species were detected either by 16S rRNA gene analysis or by MALDI-TOF MS except for the opportunistically pathogenic bacterium *Pseudomonas aeruginosa*. In addition, in house software that calculated the correlation coefficient values (CCV) of the mass spectral raw data and their variation was developed and used to allow the rapid and efficient identification of marine bacteria in ballast water for the first time.

## Introduction

Shipping moves over 80% of the world’s commodities and transfers approximately 3–5 billion tons of ballast water around the world every year. Ships’ ballast tanks hold different non-indigenous vertebrates, invertebrates, plants and microorganisms [1]–[3]. Thus, marine bio-invasion by ballast water derived organisms may threaten naturally evolved biodiversity, the consequences of which are being increasingly realized in recent years [4]. The microbiology of ballast water is also of increasing environmental importance, since discharged ballast water may contain infectious pathogens and may thus lead to their global dissemination [1]. Currently monitoring and characterization of marine bacteria in large volumes of sea water is time consuming and costly. To make this process faster, cost-efficient and more reliable, we are in the process of constructing comprehensive MALDI-TOF mass-spectral libraries of bacterial species from ballast water samples to allow tailored and rapid processing to be carried out.

In order to restrict aquatic invasions, The IMO has established guidelines for the control and management of ballast water to minimize the transfer of harmful aquatic organisms and pathogens [5]. IMO regulation D-2, ‘Ballast Water Performance Standard’ states that indicator microbes in water discharged by ships shall not exceed specified concentrations [6]. These include, but are not limited to: *Vibrio cholera*, *Escherichia coli*, and intestinal enterococci. In 1975, Anhalt and Fenselau first applied mass spectrometry to bacterial identification [7]. After that, MALDI-TOF MS Biotyping (MTB) solely or together with other proteomic tools has become a common technique for detection, identification, and characterization of micro-organisms [7]–[10]. This method can be complementary to the acquisition of data obtained from 16S rRNA gene sequencing with the added benefit of generating unique biochemical fingerprints for the sub-typing of species.

There are few reports of the use of MTB to identify bacteria in large volumes of water [11]. However, liquid chromatography (LC)/MS/MS has been used not only for identification of bacteria, but also for characterization of the toxins produced by some species in water. Dworzanski and co-investigators have applied LC/MS/MS with statistical tools for identification of bacteria including *Bacilllus anthracis* [12]–[14]. LC/MS/MS for characterization of cyanobacterial toxins produced in Italian lakes has also been reported [15]–[17].

A major limiting factor in the use of MTB for characterization of aquatic bacteria is the lack of a suitable mass spectra database for rapid and reliable species identification during processing. Most currently available databases are focused on clinically important species associated with human disease. In the present study, Biotyper 2.0 software (Bruker Daltonics) was employed, which currently contains ∼4000 reference mass spectra. However, most of the mass spectra deposited in this library belong to terrestrial organisms and mass spectra for some important groups of marine bacteria such as members of the genus *Pseudoalteromonas* are not included.

Although both the intact bacterial cells and crude cell lysates can be used for MTB, a key to successful characterization of bacteria by this method is the experimental consistency of the working material. Lasch et al. [18] described a proteomic technique that uses intact cell MS for reproducible detection of microbial protein patterns to complement genotypic or phenotypic testing methods. In contrast, in the current study, bacterial lysate profiling was employed, which resulted in mass spectra of intra-cellular proteins without the need for separation of cellular components. In our experience, the quality of the spectra obtained from lysates was higher than those from intact cell protein fingerprints (data not shown). This also in line with work by Böhme et al on MTB of food borne bacteria, who observed less noise and relatively more reproducibility when using cell lysates compared to data obtained from intact cells [19]. In addition, in the case of isolation of potentially pathogenic contaminants, the MALDI-TOF instrument and accessories would not be contaminated since microbiologically sterile lysates would be used instead of whole cells. In-house software that calculated the correlation coefficient values (CCV) of MS raw data and their variations was developed. We used correlation analysis to determine inter- and intra-specific similarities of bacterial isolates and successfully compared characteristic bacteria isolated in this study. The method was validated by comparing the MS results with the taxonomic identification obtained by 16S rRNA gene sequence analyses.

## Materials and Methods

### Preparation of Artificial Ballast Water

Seawater was collected from the Dove Marine Laboratory, Newcastle University, Cullercoats, North Tyneside (55°02′00″ N, 001°27′00″W) at low tide, from a depth of 2 m and also from Blyth harbour (55°08′14″N, 001°31′51″ W) at low tide at a depth of 2m, 100m from the coastline. Seawater was pumped from Cullercoats bay into 55000 litre storage tanks and stored outdoors. Samples (1l) were removed and filtered to collect bacteria. The used tanks mimicked conditions in ballast tanks. They were steel lined, entirely sealed and did not allow any air exchange or light to enter.

### Bacterial Strains and Culture Conditions

Surfactant-free cellulose acetate filters with a 0.2-µm pore size (Filtropur V filter system, Starstedt Ltd.) were used to separate bacteria from the seawater; cells were washed from the filters with 2 ml sterile seawater. Although by filtering, some bacteria may be left attached, this method was chosen for its speed and ease of later automation. Serial dilutions were made from aliquots of 100 µl of the bacterial suspension and cultured. A1 medium [20] at room temperature was used for isolation of coliforms, *Pseudomonas* isolation-agar [21] at 37°C for pseudomonads, M-*Enterococcus*-agar [22] at 37°C for enterococci; and Thiosulfate Citrate Bile Salts Sucrose-agar (TCBS) [23] at room temperature for isolation of vibrios. Following bacterial growth on selective media, single colonies were isolated and sub-cultured on seawater agar twice in order to obtain pure cultures, and remove culture medium as a source of variability. Seawater-agar was prepared by dissolving the following components in 500 ml distilled water: 14 g NaCl, 0.385 g KCl, 0.8 g CaCl_2_, 2.4 g MgCl_2_, 0.055 g NaHCO_3,_ 1.75 g MgSO_4_, 5 g beef extract, and 5 g peptone. Agar was added to give a final concentration of 15 g L^−1^ to solidify the media. Single colonies then were subjected to 16S rRNA gene sequencing and MTB. All selective media were purchased from Difco™ (Oxford, UK).

### 16S rRNA Sequencing and Analysis

DNA isolation, amplification, and cycle sequencing of samples from the marine bacteria collected in this study was performed by Geneius Laboratories Ltd (Newcastle upon Tyne, UK). Amplification of the full 16S rRNA gene was carried out as described by Lane, 1991 [24]. The resulting rRNA gene sequences were then used to search the data available at the National Center for Biotechnology Information (NCBI, http://blast.ncbi.nlm.nih.gov/Blast.cgi) using the basic local alignment search tool (BLAST) algorithm. The closest bacterial species identified through nucleotide matching was considered to be the potential microorganisms of interest. Sequences generated during this study are available from the DNA Data Bank of Japan (DDBJ). Accession numbers for the 16S rRNA gene from each isolate are listed in **Table 1** and are also shared with NCBI (National Resource for Molecular Biology Information) and EMBL (European Molecular Biology Laboratory). The phylogram of *Vibrio* species was generated by using the ClustalW (http://www.ebi.ac.uk/Tools/msa/clustalw2/) program from neighbour analysis of approximately 1500 nucleotides.

**Table 1:** Identification of marine bacteria from artificial ballast water.*.

IC	Biotyper software ID	BS	16S rRNA gene ID	PL	%DNA	Accession
01-B	*Vibrio fluvialis* *CCM 3695 CCM*	1.284	*Vibrio rumoiensis*	−	95.7	AB607131
03-W	*Pseudomonas gessardii* *CIP 105469 HAM*	2.128	*Pseudomonas synxantha*	+	99.7	AB607132
04-W	*Pseudomonas stutzeri* *040_W09 NFI*	2.386	*Pseudomonas stutzeri*	+	99.6	AB607133
05-W	*Vibrio fortis LMG* *21557 HAM*	1.543	*Vibrio lentus*	+	99.4	AB607134
06-W	*Pseudoalteromonas sp.*	1.600	*Pseudoalteromonas* *marina*	–	99.7	AB607135
07-W	*Pseudoalteromonas tetraodonis*	2.845	*Pseudoalteromonas tetraodonis*	–	98.1	AB607136
10-B	*Pseudoalteromonas sp.*	1.517	*Pseudoalteromonas sp.*	–	98.1	AB607137
11-W	*Pseudomonas putidaDSM 50198 HAM*	2.123	*Pseudomonas putida*	+	98.2	AB607138
12-W	*Pseudomonas mendocina DSM 50017T HAM*	2.240	*Pseudomonas oleovorans*	+	99.2	AB607139
13-W	*Vibrio tasmaniensis* *DSM 17182 HAM*	2.087	*Vibrio splendidus*	+	99.7	AB607140
14-W	*Vibrio gigantis LMG* *22741 HAM*	2.104	*Vibrio cyclitrophicus*	+	99.5	AB607141
15-W	*Enterococcus faecalis* *DSM 6134 DSM*	2.360	*Enterococcus hirae*	+	99.6	AB607142
16-W	*Enterococcus faecium 11037 CHB*	2.502	*Enterococcus faecium*	+	99.9	AB607143
17-W	*Vibrio alginolyticus* *CCM 7037 CCM*	2.167	*Vibrio rotiferianus*	+	99.0	AB607144
20-W	*Pseudoalteromonas sp.*	1.626	*Pseudoalteromonas undina*	–	99.0	AB607145
21-B	*Serratia liquefaciens* *DSM 30066 DSM*	2.062	*Serratia plymuthica*	+	99.8	AB607146
22-B	*Pseudomonas monteilii DSM 14164T HAM*	2.288	*Pseudomonas fulva*	+	98.9	AB607147
23-B	*Pseudomonas tolaasii* *LMG 2342 HAM*	2.110	*Pseudomonas tolaasii*	+	99.4	AB607148
24-B	*Proteus vulgaris DSM 13387 HAM*	2.024	*Pseudomonas stutzeri*	+	99.2	AB607149
25-B	*Pseudomonas alcaligenes DSM 50342T HAM*	1.432	*Pseudomonas stutzeri*	+	98.0	AB607150
27-B	*Vibrio pomeroyi DSM 17180 HAM*	2.317	*Vibrio tubiashii*	–	95.5	AB607151
28-B	*Halomonas aquamarina DSM 4739_DSM*	1.568	*Halomonas venusta*	+	99.7	AB607152
29-B	*Halomonas halmophila B510_UFL*	1.452	*Idiomarina loihiensis*	–	99.5	AE017340
30-W	*Vibrio cyclitrophicus* *LMG 21359 HAM*	2.168	*Vibrio cyclitrophicus*	–	98.0	AB607154
32-B	*Vibrio gigantis LMG* *22741 HAM*	2.116	*Vibrio tubiashii*	–	95.7	AB607155
33-W	*Serratia plymuthica* *DSM 4540_DSM*	2.185	*Serratia plymuthica*	+	99.9	AB607156
34-W	*Pseudoalteromonas sp.*	1.324	*Pseudoalteromonas sp.*	–	99.7	AB607157
36-B	*Pseudoalteromonas sp.*	2.519	*Pseudoalteromonas atlantica*	–	98.0	AB607158
37-B	*Pseudomonas brenneri* *DSM 106646T HAM*	2.113	*Pseudomonas synxantha*	+	99.7	AB607159
38-B	*Pseudomonas stutzeri 040_W09 NFI*	2.240	*Pseudomonas stutzeri*	+	99.9	AB607160
39-B	*Pseudoalteromonas sp.*	1.927	*Pseudoalteromonas carrageenovora*	–	99.4	AB607161
40-B	*Tenacibaculum discolor NCIMB 14278 EGS*	2.048	*Tenacibaculum sp.*	+	99.5	AB607162
41-B	*Bacillus mycoides DSM 2048_DSM*	2.359	*Bacillus mycoides*	+	99.6	AB607163
42-B	*Vibrio alginolyticus DSM 2171 HAM*	2.240	*Vibrio natriegens*	+	97.0	AB607164
43-B	*Bacillus hwajinpoensis DSM 16206_DSM*	1.746	*Bacillus baekryungensis*	–	99.8	AB607165
44-B	*Enterococcus hirae DSM 3320_DSM*	2.410	*Enterococcus hirae*	+	99.9	AB607166
45-B	*Lactobacillus paraplantarum DSM 10641_DSM*	1.750	*Lactobacillus pentosus*	+	99.8	AB607167
49-B	*Comamonas testosteroni B337 UFL*	1.333	*Pseudoalteromonas carrageenovora*	–	99.9	AB607168
50-B	*Pseudomonas aeruginosa 19955_1 CHB*	2.270	*Pseudomonas* *aeruginosa*	+	99.8	AB607169

**IC**: isolate code. **B**: Blyth; **W**: Whitley bay; **BS**: Biotyper software score.The score ranged from 0–3, scores >1.7 indicates a positive genus ID, scores <1.7 mean no reliable identification; **PL** Presence of the species in Biotyper mass spectra Library, (+): presence and (−): absence. % DNA indicates the % of rRNA gene nucleotides matching of the query and publically available sequences at NCBI by BLAST search. Accession numbers were from DDBJ.

### Sample Preparation for MALDI-TOF MS Analysis

Two to five single colonies of actively growing cultures were used in sample preparation. An even bacterial suspension was made in 300 µl of double-distilled water which was then fixed by the addition of 900 µl absolute ethanol. The samples were centrifuged (13000 g, 5 min) and the supernatant was completely removed under vacuum. Lysates were prepared by adding 50 µl 70% formic acid to the bacterial pellet and mixing thoroughly, before adding 50 µl acetonitrile and again mixing thoroughly. Following centrifugation (13000 g, 2 min), the supernatant was transferred to a fresh tube, and then 1 µl of the supernatant containing the bacterial lysate was transferred to a sample position on a 384 ground steel MALDI target plate (Bruker Daltonics, Coventry, UK) and air-dried at room temperature. The sample was overlaid with 1 µl of MALDI matrix and again air-dried.

### Preparation of Matrix for MALDI Analysis

A saturated solution of α-cyano-4-hydroxy-cinnamic acid (HCCA, Bruker Daltonics) in 50% acetonitrile: 2.5% tri-fluoro-acetic acid was prepared by adding 1 ml of the organic solvent to 10 mg of HCCA, and subjecting the mixture to sonication in a water bath (Ultrasonic Cleaner HF45kHz/60W, VWR™) for 15 min before centrifugation at 13000 g for 2 min. The supernatant was used as the matrix for crystallizing the protein samples.

### MALDI-TOF Parameters

For database construction and validation, measurements were performed in the auto execute mode using an UltraFlex II mass spectrometer (Bruker Daltonik, Leipzig, Germany) with fuzzy control of laser intensity and a 1∶25 kV; Ion source 2∶23.5 kV; laser frequency: 50.0 Hz; detector gain, 1650 V; and gating, maximum, 1500 Da. Spectra were recorded in the positive linear mode for a mass range of 2000 to 20000 Da. Each spectrum was obtained by averaging 600 laser shots acquired from the automatic mode under control of flexControl software V. 3 (Bruker Daltonics). The spectra were externally calibrated using an *E.coli* DH5-alpha standard (Bruker Daltonics). The calibrant consisted of seven ribosomal proteins from *E. coli* with added RNAse A and myoglobin to cover a range of 3637.8 to 16957.4 Da. Biotyper 2.0 software (Bruker Daltonics) was used for the identification of bacterial isolates.

### Generation of Reference Mass Spectra for the Genus *Pseudoalteromonas*

Some of the bacterial isolates in this study could not be identified even at the genus level using MTB. However, 16S rRNA gene sequencing revealed them to belong to the genus *Pseudoalteromonas*. Biotyper 2.0 software was lacking MS data for this genus. For example, isolate S07 was detected as *Pseudoalteromonas* with 98% nucleotide matching to that of *P. tetraodonis*. The spectra from this isolate was accepted and added to the Biotyper 2.0 mass spectra library as *Pseudoalteromonas sp.* After re-analyses of the data many of the unknown isolates could be identified as *Pseudoalteromonas*. In addition *Pseudomonas* and *Pseudoalteromonas* isolates were employed to test the efficiency of in-house software in discrimination of bacterial species. The method for adding the spectra to the database was similar to the protocol described by Freiwald and Sauer [25]. Briefly, isolate S07 was subjected to protein preparation as described above. Then bacterial cell lysate from freshly grown single colony was used for the analysis. Data were collected from the sample that was applied to 8 different spots on a 384 ground stainless steel target plate. Each spot was read 4 times, 1000 shots each. The quality of all spectra was inspected simultaneously under flexAnalysis control. After smoothing and baseline subtraction, averaged mass spectra for *Pseudoalteromonas* was created via selection of 20 spectra by eliminating those with higher deviation, and then added to the unassigned Biotyper library.

### In-house Matlab Based Correlation Coefficient Value (CCV) Analysis

One approach to pattern identification within a mass spectral signal uses cross correlation of the mass spectra signal with a known mass spectrum in a library. Where the library mass spectra signal and the unknown signal are similar the cross correlation will be high. A program for Matlab (MathWorks Inc.,Cambridge, UK), was therefore developed to calculate the Correlation Coefficient Value (CCV) from the mass spectra raw data. The correlation coefficient defines the dependence structure between the two sets of data. Correlation coefficients were calculated for more than two sets of data. In this case the data set was an array X, in which each row was an observation at a certain mass-to-charge ratios (*m/z*) and each column was a variable (mass spectra intensity). If C is the covariance matrix, then CCV elements in the correlation coefficient matrix were calculated using following equation:
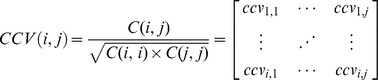



In order to compare the MALDI-TOF mass spectra of some isolates, a correlation coefficient matrix (CCM) with CCV elements was calculated. The CCM was also used for the first inspection of relationships among the variables of the multivariate data sets. The collected data were standardized (mean subtracted and normalized) before any further calculations were performed. The CCM was inspected for any outliers that could result in an incorrect correlation, and then any non-overlapping spectra from a set of 30 spectra generated by MALDI-TOF, for each isolate were deleted.

## Results and Discussion

An objective of this study was the rapid detection of specific bacterial genera in ballast water. Two isolates (S29 and S49) could not be identified by MTB due to lack of information in the initial reference library. Nevertheless, S49 mass spectra showed high similarity to the mass-spectrum of *Pseudoalteromonas sp*. This data was added to the Biotyper 2.0 library. The marine bacteria characterized in this work are listed in **Table 1**.

### *Vibrio* Isolates

**Figure 1:**
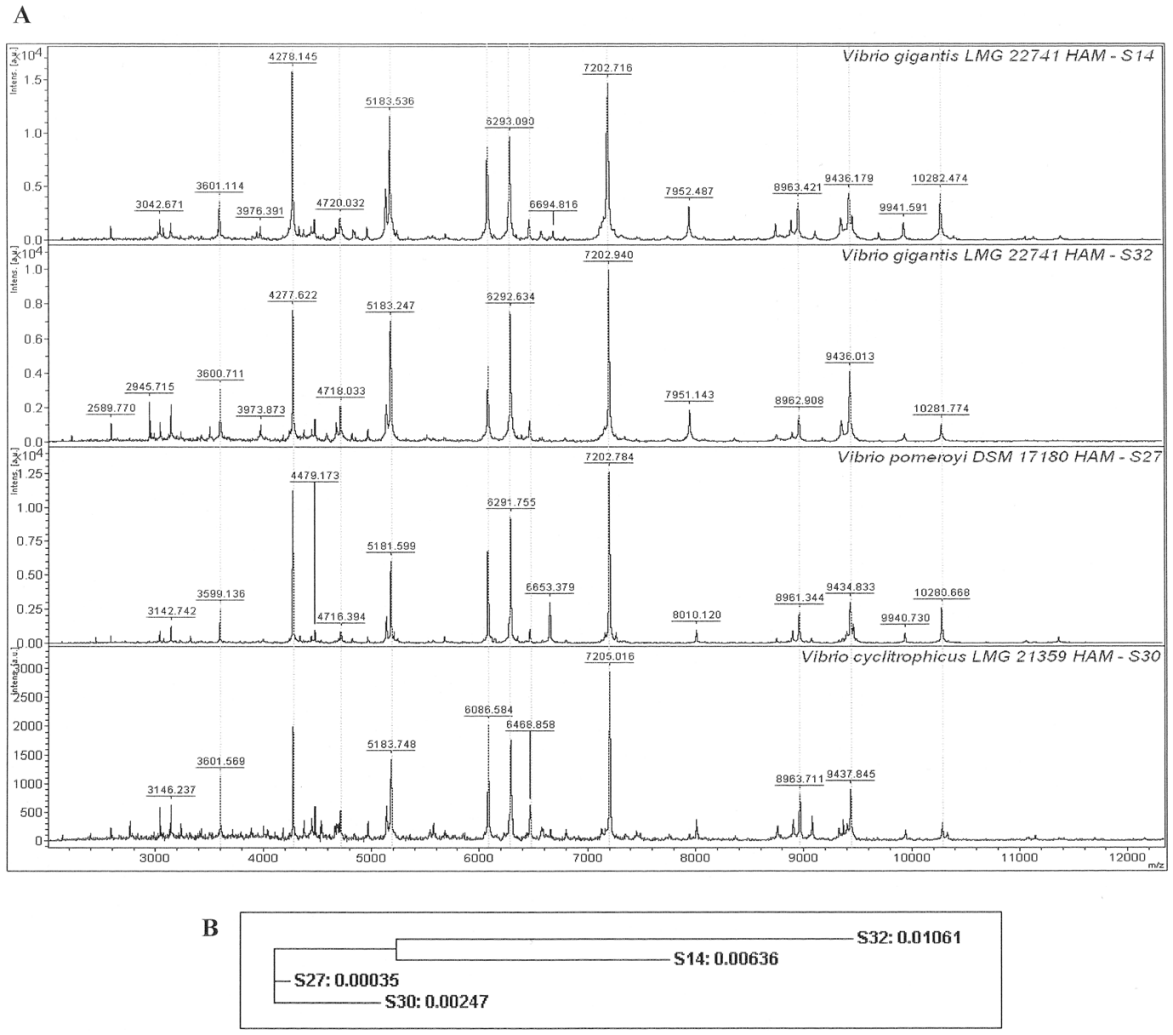
MALDI-TOF mass spectrograms, (panel A) and 16S rRNA gene sequence phylogram, (panel B) for four *Vibrio* isolates from the North Sea. Panel A, the X axis indicates the mass to charge ratios (*m/z*) of each peak and the Y axis for each individual plot indicates intensity of the peaks. The identification result using Biotyper software is shown at the top-right corner of each plot followed by the type strain designation. Several mass spectral peaks are common between the isolates and a few show differences, examples of similar peaks are aligned by dotted lines. Panel B, distance phylogram tree of the isolates, over 1500 nucleotides.

*Vibrio* species are commonly found in seawater and are of special interest due to their potential to cause specific diseases, such as gastroenteritis, cholera, sepsis, and cellulitis leading to diarrhea and necrotizing soft tissue infections [26], [27]. Due to increased global marine traffic, the spread of potentially harmful *Vibrio* species deserves particular attention on shores of highly populated European countries [28]. IMO is also specifically concerned about the concentrations of serotypes O1 and O139 of *V. cholerae* in ballast water [6]. *V. cholerae* was not detected in this study. Four out of eight *Vibrio* isolates in this study, show high similarities at the nucleotide and protein levels. MTB analysis of the isolates S32, S27, S14 and S30 by FlexAnalysis software revealed that they all contain major peaks with similar *m/z* and relative intensities. MS data of isolates S14, S32, and S27 followed by Biotyper software analysis supported the possibility that isolates S14 and S32 belonged to the same species, *V*. *gigantis* (**Fig. 1**). By comparing the 16S rRNA gene sequences of all four *Vibrio* isolates using the ClustalW2 alignment program, it was found that the differences were not significant enough to confidently differentiate them into separate species (**Table 2**). 16S rRNA gene comparison and BLAST search however implied that isolates S27 and S30 are similar to *V*. *cyclitrophicus*. Isolate S30 was also identified as *V. cyclitrophicus* by MALDI-TOF analysis and Biotyper software. However, the identity of the nucleotide sequence of S30 to that of the type strain of *V. cyclitrophicus* in NCBI database was only 98%. Likewise, relatively low Biotyper score of 2.1 out of 3.0 indicated uncertainty in the species detection by both methods. There were few differences in mass-spectra of the four above mentioned isolates, for example, the mass spectrum of isolate S27 contained peaks at *m/z* 8010 and 6653, which were missing in isolates S14 and S32. Peaks at *m/z* 3973 and 3976 in isolates S32 and S14 were missing in isolate S27 (**Fig. 1**). Although some of the differences between closely related species may be due to amino acid substitutions, the *m/z* differences between these isolates did not indicate any altered amino acid sequences.

**Table 2:** Mass spectral peak list comparing three marine bacterial species isolated in this study with previously published data.*.

*S. liquifaciens*			*V. alginolyticus*			*P. vulgaris*		
This study	Previous data [30]	±*m/z*	This study	Previous data [37]	±*m/z*	This study	Previous data [37]	±*m/z*
2171	2175	4	3149	3150	1	3135	3134	1
2695	2698	3	3595	3596	1	3635	3636	1
3118	3120	2	4046	4045	1	4482	4483	1
3608	3603	5	4277	4276	1	4735	4735	0
–	3960	–	4536	4531	5	5128	5129	1
4182	4183	1	4703	4703	0	5507	5510	3
4348	4347	1	4724	4725	1	6265	–	–
4638	4640	2	5139	5138	1	6499	–	–
4780	4780	0	5179	5178	1	7269	7272	3
5154	5155	1	5522	5523	1	7810	7808	2
5396	5395	1	6407	6405	2	8363	8364	1
5534	5533	1	7194	7191	3	8826	8827	1
6016	6016	0	8088	8089	1	9469	9470	1
6060	–	–	8755	8753	2			
6240	6237	3	9451	9447	4			
6452	6451	1						
7299	7298	1						
–	7667	–						
7920	7918	2						
8105	8105	0						
8861	8859	2						
8979	–	–						
9280	9280	0						
9564	9558	6						

* MALDI-TOF mass spectra peak list for *Serratia liquifaciens*, *Vibrio alginolyticus* and *Proteus vulgaris* obtained in this study and from data previously published [30], [37]. Mass to charge ration (±*m/z*) column indicates mass unit differences between the two studies and (–) indicates no data were available.

A summary of the nucleotide sequence comparisons is shown in **Figure 1B**. Species identification using 16S rRNA gene sequence analysis is not always reliable. Therefore, detailed discrimination of this group of bacteria requires more detailed analysis through polyphasic approaches. In a study by Hazen et al. [29], MALDI spectra of 10 *Vibrio* isolates were compared. The authors employed an MTB approach since many *Vibrio* species such as *V. alginolyticus*, *V. parahaemolyticus*, *V. harveyi*, and *V. campbelli,* are indistinguishable by 16S rRNA gene analysis. Interestingly, although Hazen and colleagues found a major peak of approximately *m/z* 12000 in all of the isolates tested, such a peak was missing in spectra from species that were collected from the North Sea (**Fig. 1**). This might be due to different materials used within these two studies. For instance, the matrix in the Hazen et al. study was sinapinic acid (SPA) which mainly favors larger proteins, whereas in current study the matrix compound was HCCA. Hazen et al. also noticed variation in the spectral patterns of samples isolated at different geographical locations at different times.

**Figure 2:**
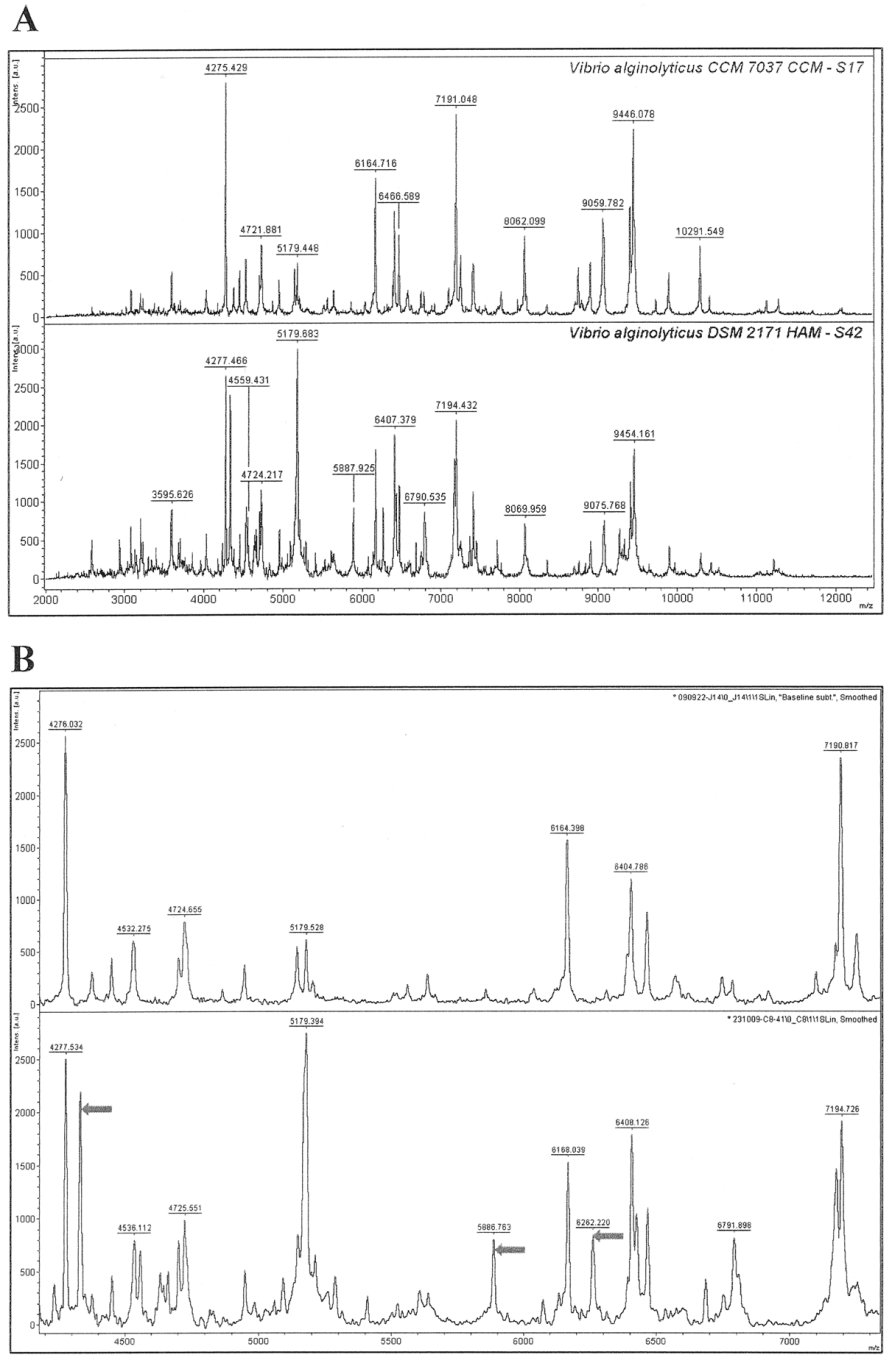
MALDI-TOF spectra of two isolates of *Vibrio*, S17 and S42. Although Biotyper software identified these isolates as strains of *V*. *alginolyticus*, they were identified as *V. rotiferianus* and *V*. *natriegens* respectively by 16S rRNA gene BLAST search. Panel A shows the spectra for the range *m/z* 2,000–20,000 and panel B is an enhanced view of the *m/z* 4,000–7,500 range. The X axis indicates the mass-to-charge ratios (*m/z*) and the Y axis in each individual plot indicates relative intensities of the ions. Examples of peaks that are not common between the two isolates are indicated by arrows.

**Figure 3:**
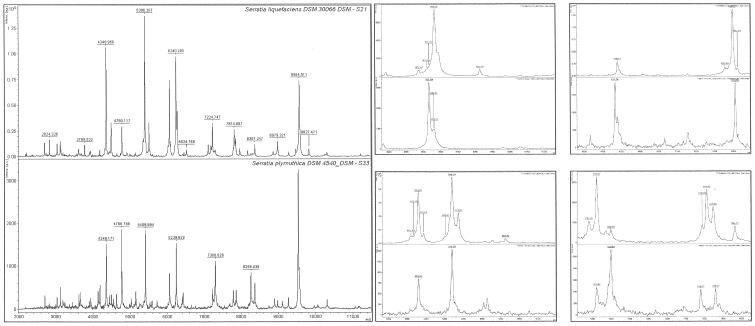
MALDI-TOF mass spectra of two *Serratia* species. 16S rRNA sequencing indicated that isolates S21 and S33 were both *Serratia plymuthica*. However there are differences in their mass-spectral patterns. The whole *m/z* 2,000–20,000 spectra of the two isolates are shown in the main panel (left). The small panels show highlighted regions of the spectra with more significant the mass-to-charge ratio (*m/z*) differences.

Böhme et al. [30] used MALDI-TOF MS spectra for the characterization of seafood-associated bacterial species including *Proteus, Pseudomonas, Vibrio* and *Serratia.* Although Böhme et al. used a different sample preparation method in their study, there were common peaks between their *Vibrio alginolyticus* isolate and isolates S17 and S42 from the North Sea at *m/z* 3595, 4277, 4725, 5179, 7191, and 9446. (**Fig. 2**, **Table 2**). Interestingly, in the same study, Böhme and colleagues generated a *Serratia liquefaciens* mass spectrum that showed similarities with the protein fingerprints of the isolate of the respective bacterium from the ballast water obtained herein. There were for instance peaks at *m/z* 4347; 4780; 5395; and 6237 (**Fig. 3**, **Table 2**).

Dieckmann et al. [31] also developed species-specific bio-markers and characterized several *Vibrio* isolates. They concluded that MTB and *rpoB* gene sequencing generated comparable classification results. The authors used SPA for data acquisition by an Ultraflex TOF-TOF MS. Interestingly, out of six species-specific biomarkers detected for *V. alginolyticus*, there were three peaks identical to those of *V. alginolyticus* strains S17 and S42 (at *m/z* 6168; 6407; and 8755). Three additional peaks showed high similarity with only a *m/z* ±2 difference (*m/z* 4277; 5179; and 6466). In other work to differentiate *Vibrio* isolates, Oberbeckmann et al. [26] chose a polyphasic approach. They employed 16S rDNA and *rpo*B sequencing in addition to MTB analysis and found that 47 tested isolates showed more than 80% similarity. However, MTB also showed less consistency in grouping the isolates, than the genomic approach [26]. Differentiation of closely related isolates of *Vibrio* therefore requires a multidisciplinary approach, since techniques such as 16S rRNA sequencing alone are inadequate. As part of multidisciplinary approaches MTB provides invaluable information that assists discrimination of closely related species.

### *Enteroccocal* Isolates

**Figure 4:**
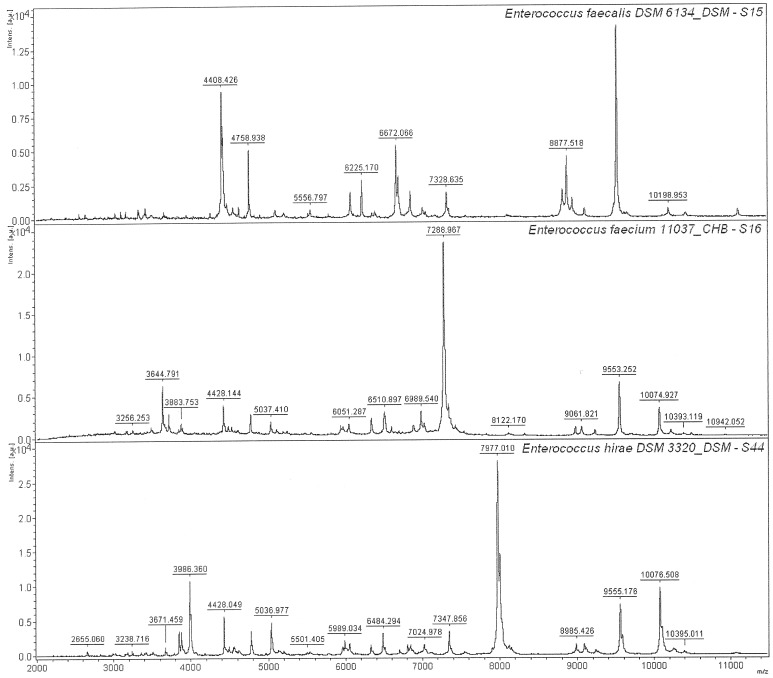
MALDI-TOF mass spectra for *Enterococcus* isolates. 16S rRNA gene analysis indicated that isolates S15 and S44 are the same species (*E. hirae*). However the protein fingerprints were not quite the same. The identification result using Biotyper software is shown at the top-right corner followed by the strain designations.

Important clinical infections are caused by some *Enterococcus* species including urinary tract infections, diverticulitis, and meningitis [32]. IMO regulations have specified that the concentration of intestinal enteroccoci in discharged ballast water should not exceed 250 cfu 100 ml^−1^ [6]. In our study, three isolates from the North Sea were identified as species of *Enterococcus* (**Table 1**). Using 16S rRNA gene sequences, isolates S15 and S44 were both identified as *E. hirae*. However, using the MTB method, only isolate S44 was identified as *E. hirae* while isolate S15 was found to be *E. faecalis*. **Figure 4** shows clear differences between the protein fingerprints of these two isolates. Additionally, there are more similarities between the mass spectra of isolates S16 and S44 than between isolates S44 and S15. This is an example of the higher discriminatory power of MTB over 16S rRNA gene analysis. The approach used here also demonstrated that the sample of ballast water used would have passed IMO regulatory restrictions.

### *Pseudomonas* and *Pseudoalteromonas* isolates

**Figure 5:**
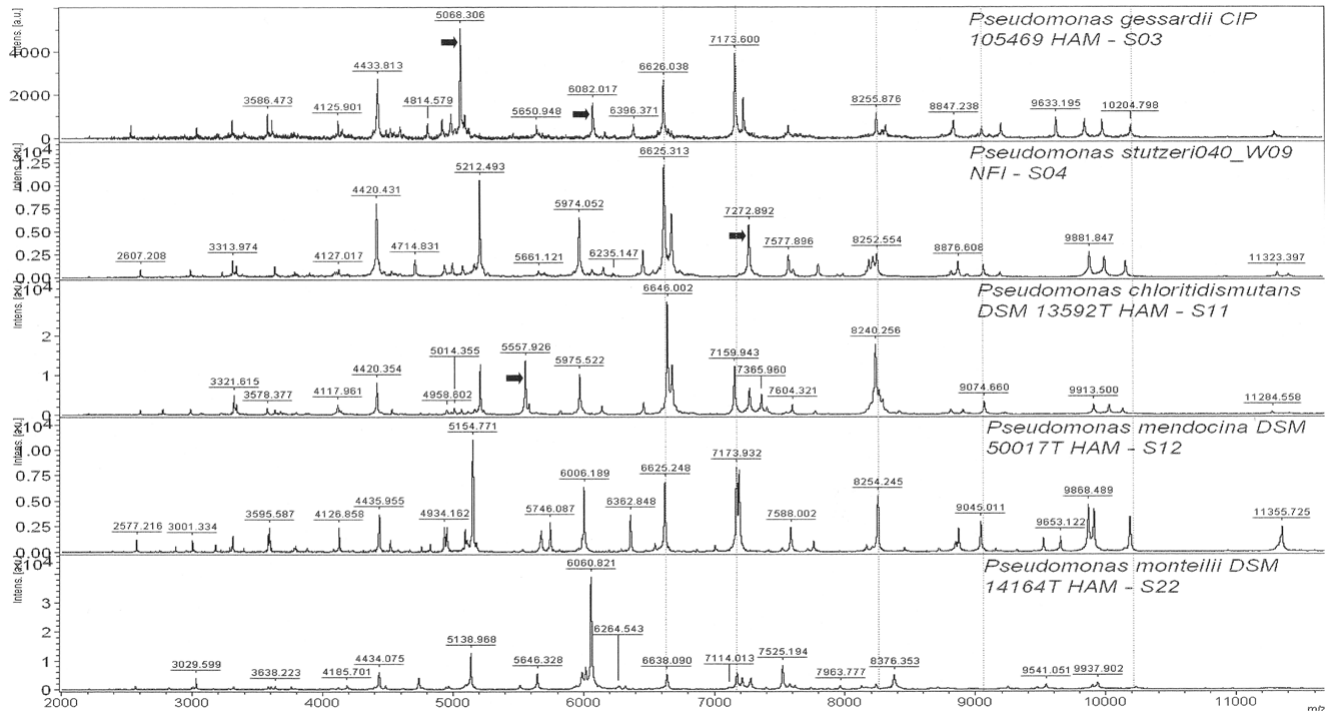
MALDI-TOF mass spectra of five *Pseudomonas* isolates. Species of *Pseudomonas* are distinguishable by their protein fingerprints. Examples of the peaks that are common between species are shown by dotted lines; peaks that can be used for differentiation of the isolates are indicated by arrows.

**Figure 6:**
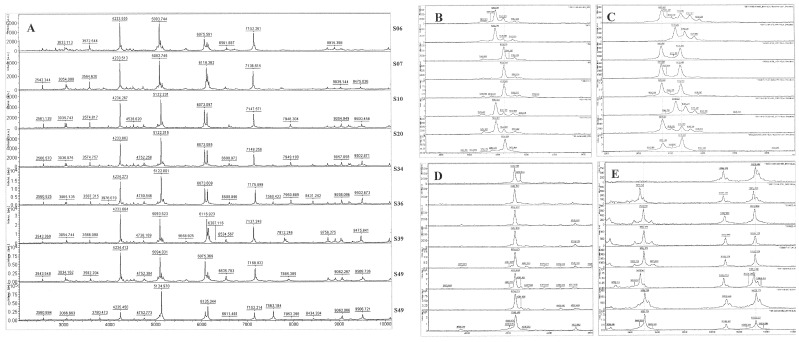
MALDI-TOF mass spectra from *Pseudoalteromonas (PAL)* isolates. The mass spectrum of *P. tetraodonis* was added to the database after identification of an isolate by 16S rRNA gene sequencing. Isolate S49, with some similarities to the mass spectral pattern of *PAL* is also included in the comparisons. Panel A shows spectra of all *PAL* isolates for the range of *m/z* 3000–10000. Panels B to E show similarities and differences between the *PAL* isolates at different *m/z*. The order of isolates in all panels is as labelled in panel A.

Members of the genus *Pseudoalteromonas* were initially described as belonging to the genus *Pseudomonas* in 1963 [33]. In 1995, Gauthier et al. [34] by describing 13 species which previously were identified as *Alteromonas*, proposed a new genus *Pseudoalteromonas*. Pseudomonads as well as some *Pseudomonas*-like species are widespread throughout nature and some such as *P*. *aeruginosa* are opportunistic pathogens of humans and plants [35]. *Pseudomonas*-like species that were isolated in this study are listed in **Table 1**. MALDI mass-spectra from pseudomonads are presented in **Fig. 5** while *Pseudoalteromonas* isolates are shown in **Fig 6**. Dieckmann et al. [36] identified *Pseudomonas*-like bacteria present in marine sponges by MS but found ambiguities both at the inter- and intra-species level when comparing 16S rRNA gene sequences. They observed that for species of *Pseudoalteromonas* and *Alteromonas* inter-species diversity had been as high as intra-species diversity. In the former study, some *Pseudoalteromonas* isolates differed by only 3 or 4 nucleotide positions over the ∼1500 bp of their 16S rRNA gene sequence but were easily discriminated by MTB. Dieckmann et al. [36] used different instruments and chemicals in their study. For example, they obtained data by running the samples on a VOYAGER mass spectrometer (Applied Biosciences). Moreover, the bacteria were grown on different media at different temperatures. Finally, the sample preparation method and matrix (2, 5-Dihydroxybenzoic acid; DHB) used were not the same. Nevertheless, there were significant similarities between their characteristic *Pseudoalteromonas* mass spectra and the spectra we obtained for the corresponding isolates. For example, we observed peaks at *m/z* 4233±2 while they observed one at 4238. In four isolates of the ballast water a peak at *m/z* 5093±1 was detected, and the former authors measured one at *m/z* 5094 in two out of three of their *Pseudoalteromonas* isolates. For five of our *Pseudoalteromonas* isolates, we identified a peak at *m/z* 6072±3 and they also found a peak at *m/z* 6074. Isolates S06 and S49 showed a peak at *m/z* 7152 that was close to the peak of *m/z* 7151 observed in the Dieckmann et al. study. These results indicate the robustness of the MTB method across different laboratories and conditions.

**Figure 7:**
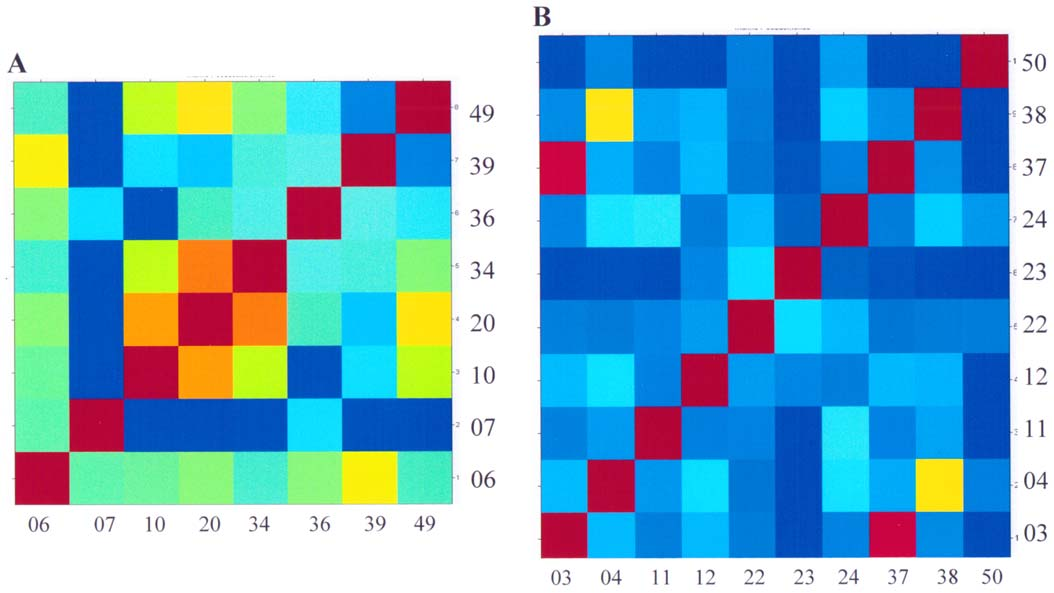
Heat-map of *Pseudoalteromonas* (A) and *Pseudomonas* (B) isolates generated by the Biotyper software. The spectra were split into 8 or 11 groups according to the directory structure. Red colours indicate closely related groups with identical peaks, and blue colours mark non-related groups.

Since the current Biotyper 2.0 database did not contain *Pseudoalteromonas* spectra and following the identification of isolate S07 by 16S rRNA sequence as *Pseudoalteromonas tetraodonis*, the spectrum of this isolate was added to the library as *Pseudoalteromonas. sp*. Consequently, when re-using the Biotyper software some of the previously unknown isolates could be subsequently identified as *Pseudoalteromonas sp*. (**Table 1**). A genus-specific peak of *m/z* 4233±2 was found in all isolates of this group (**Fig. 6**, panel **D**). Nearly all ballast water *Pseudoalteromonas* isolates had peaks at *m/z* 5092±2, *m/z* 6072±3, and *m/z* 5120±4 (**Fig. 6**). However, the *m/z* 7100 zone could also be used to discriminate these isolates. Isolates S07 and S36 showed high similarities in their mass-spectra patterns and are therefore likely to be strains of the same species. Isolates S20 and S49, which were related to *Pseudoalteromonas* by 16S rRNA gene analysis, could not be robustly identified using MTB (Biotyper score <1.7). However, these strains were included in the *Pseudoalteromonas* group since they showed high overall similarities in their mass spectral patterns with other members of the group (**Fig. 6**). An overview of the similarities of all the *Pseudomonas* and *Pseudoalteromonas* isolates is shown in **Fig. 7** as a heat-map.

### MTB Identification of a Marine *Proteus* isolate

**Figure 8:**
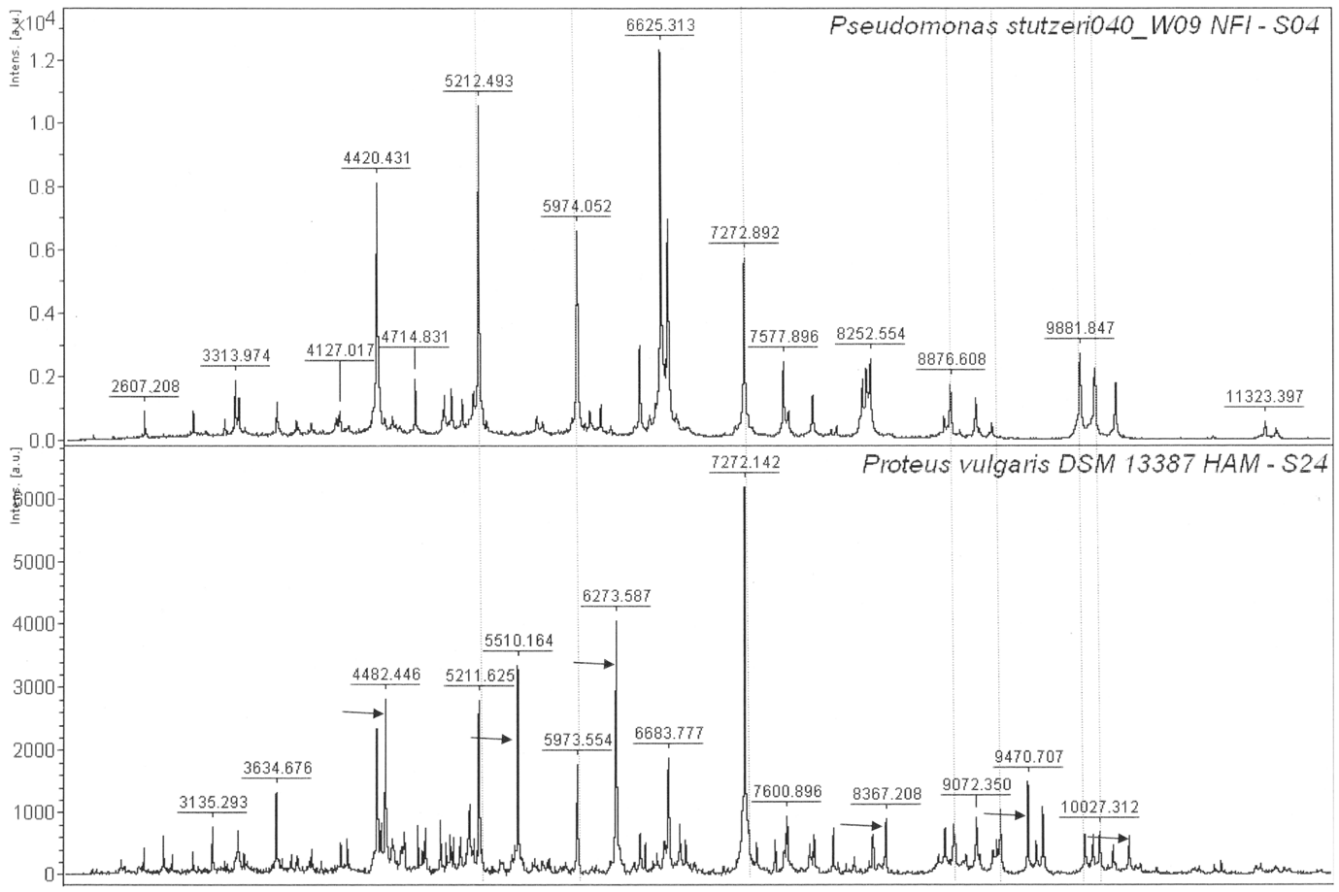
Comparison of *Pseudomonas stutzeri* and *Proteus vulgaris* MALDI-TOF mass spectra. Isolates S04, S24 and S38 were identified as *P. stutzeri* through 16S rRNA gene sequencing; however isolate S24 showed a different peak pattern and was identified as *Proteus vulgaris* by the Biotyper software. Examples of common peaks are indicated by vertical lines and unique peaks are indicated by the arrows.

Isolate S04 was identified as *Pseudomonas stutzeri* by both MTB and 16S rRNA gene sequencing. Isolate S24 was also identified as *Pseudomonas stutzeri* by 16S rRNA gene analysis whereas by MTB, this isolate was identified as *Proteus vulgaris*. The mass-spectrum of isolate S04, was compared with that of isolate S24 (**Figure 8**) showing that, although there are common peaks between the two isolates, there are also clear differences that separate them as two different genera. For example, S24 has more peaks in the higher *m/z* range than S04. They both had an *m/z* 7272 peak but the *m/z* 6624–25 peak was missing in the S24 isolate mass spectrum and peaks at *m/z* 6273 and 5510 in S24 were missing in S04. Therefore it appears that the MTB method is providing more reliable information on a strain’s identity. The mass-spectrum of isolate S24 showed relatively high relatedness to a *Proteus vulgaris* isolate in the study by Fernandez-No et al. [37] who used MALDI-TOF MS to characterize food-borne pathogens (**Table 2**, **Fig. 8**). Although the *m/z* 6265 and 6499 peaks were missing in S24, the maximum difference observed between the other 11 peaks was only ±3 Da. It is important to note that Fernandez-No et al. [38] used a different mass spectrometer (Voyager DE STR, Applied Biosystems) and followed a different protocol for sample preparation. These experimental differences on the one hand and the broad concurrence of data on the other further demonstrate the high level of reproducibility of the MTB method.

### CCV Analysis of Selected Bacterial Isolates

**Figure 9:**
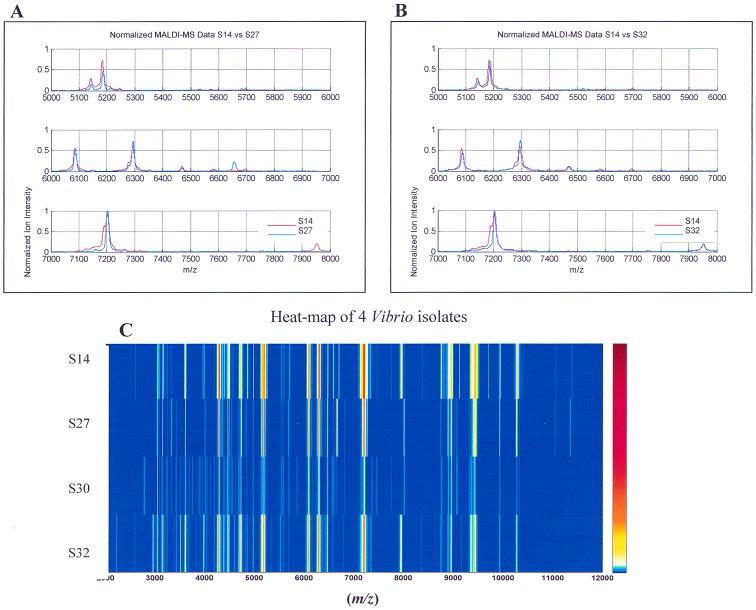
Examples of in-house CCV analysis of *Vibrio* isolates S14 and S27 (A) and S14 and S32 (B). Panel C shows the spectrograms of *Vibrio* isolates S14, S27, S30, and S32.

In order to evaluate the potential of our in-house software, mass spectra of selected isolates of *Vibrio, Pseudomonas*, and *Pseudoalteromonas* were subjected to more detailed analysis. Mass spectra of the four closely related *Vibrio* isolates S14, S27, S30, and S32, were compared by calculating a CCV matrix. MS data showed that the major peaks present in all four isolates had similar *m/z* but different intensities (see **Figs. 1** **and** **2**). Plots of normalized data for these four isolates are presented in **Table 3** and **Figure 9**, where panels A and B compare the spectrum of isolate S14 with those of isolates S27 and S32. The results shown in **Table 3** indicate that with 92% confidence *Vibrio* isolates S14 and S32 were closest to each other confirming the conclusions of the 16SrRNA gene sequences phylogram (**Fig. 1** **, panel B**) There is a high possibility that isolates S14 and S27 or S14 and S32, respectively, are identical species (CCV 0.88). However, when 12 repeats of the *E. coli* standard (Bruker Daltonics) using 600 laser shots for each sample were analyzed, inter-specific variation of the CCV was in the range of 0.9–1.0 (data not shown). Consequently, this level of variance would rule out the existence of a significant mass difference in the spectra of the *Vibrio* isolates detectable by CCV analysis.

**Table 3:** CCV analysis of MALDI-TOF data of four *Vibrio* isolates using the in-house method developed for this study.*.

Isolate	S14	S27	S30	S32
**S14**	1.0000	0.8707	0.8781	0.9276
**S27**	0.8707	1.0000	0.8517	0.9134
**S30**	0.8781	0.8517	1.0000	0.9038
**S32**	0.9276	0.9134	0.9038	1.0000

* Numbers are correlation coefficients indicating similarity of the isolates through their entire mass-spectra. Values may vary from “0” to “1”, where 1 indicates 100% identity.

CCV analysis of mass spectra from 6 *Pseudoalteromonas* and 10 *Pseudomonas* isolates resulted in differentiation of the isolates. Through data analysis using the Biotyper software and in-house mass spectra analysis tools, *Pseudomonas* isolates S03 and S37 were found to be very closely related. In addition, in-house CCV analysis indicated that overall there were more similarities between the *Pseudoalteromonas* isolates than among *Pseudomonas* isolates from the ballast water (**Tables 4** **and** **5**).

**Table 4:** In-house CCV analysis of MTB fingerprints of *Pseudoalteromonas* isolates from the North Sea.

Isolate	S06	S07	S10	S34	S36	S39
**S06**	**1.0000**	0.7069	0.5719	0.5403	0.7463	0.8879
**S07**	0.7969	**1.0000**	0.5100	0.4498	0.9351	0.7357
**S10**	0.5719	0.5100	**1.0000**	0.9146	0.5149	0.5721
**S34**	0.5403	0.4498	0.9146	**1.0000**	0.4718	0.6186
**S36**	0.7463	0.9351	0.5149	0.4718	**1.0000**	0.7327
**S39**	0.8879	0.7357	0.5721	0.6186	0.7327	**1.0000**

Values may vary from “0” to “1”, where 1 indicates 100% identity of the mass-spectral patterns.

**Table 5:** In-house CCV analysis of MTB fingerprints of *Pseudomonas* isolates from the North Sea.

Isolate	S03	S04	S11	S12	S22	S23	S25	S37	S38	S50
**S03**	**1.0000**	0.3269	0.2065	0.4243	0.1915	0.5986	0.1749	0.9606	0.1630	0.0616
**S04**	0.3269	**1.0000**	0.5662	0.2692	0.1319	0.2218	0.2340	0.3093	0.3110	0.1172
**S11**	0.2065	0.5662	**1.0000**	0.1718	0.1249	0.2408	0.1301	0.1863	0.3116	0.0668
**S12**	0.4243	0.2692	0.1718	**1.0000**	0.1844	0.2246	0.1771	0.3836	0.1150	0.0481
**S22**	0.1915	0.1319	0.1249	0.1844	**1.0000**	0.1504	0.2480	0.1863	0.0847	0.0525
**S23**	0.5986	0.2218	0.2408	0.2246	0.1504	**1.0000**	0.2021	0.6238	0.1300	0.0516
**S25**	0.1749	0.2340	0.1301	0.1771	0.2480	0.2021	**1.0000**	0.1689	0.0791	0.0455
**S37**	0.9606	0.3093	0.1863	0.3836	0.1863	0.6238	0.1689	**1.0000**	0.1442	0.0689
**S38**	0.1630	0.3110	0.3116	0.1150	0.0847	0.1300	0.0791	0.1442	**1.0000**	0.0345
**S50**	0.0616	0.1172	0.0668	0.0481	0.0525	0.0516	0.0455	0.0689	0.0345	**1.0000**

Values may vary from “0” to “1”, where 1 indicates 100% identity of the mass-spectral patterns.

There are two major factors influencing the success of MTB: sample quality and access to rich, reliable mass-spectra databases. Different researchers have used different matrices for ionization of proteins. For example, when characterizing *Salmonella* strains, Dieckmann et al. [38] found that SPA generates more satisfactory results than either DBH or HCCA. In our experience, for analysis of the cell lysates, HCCA generates more peaks than SPA that can assist the differentiation of bacterial isolates. Because we currently use Bruker software and hardware we followed preparation methods recommended by this manufacturer. This included using cell lysates from actively growing colonies and HCCA. Compared to intact cells, crude lysate profiling resulted in higher quality spectra; an important factor when trying to discriminate closely related isolates. Part of the general problem with topology and morphology of solid state MALDI MS analysis is that it can result in non-homogeneous distribution of the sample, therefore fluctuation in analyte intensities can be partially overcome by application of liquid or ionic matrices [39]. Nevertheless, there are contradictory reports on the effect of culture age and type of culture media on bacterial whole cell protein mass-spectral patterns. Pennanec et al. [40] demonstrated that age of the culture and the culture media did not result in significantly different mass fingerprint spectra. This could potentially be explained by the dominance of peaks resulting from ribosomal proteins and proteins with house-keeping functions which in general stay unchanged under different growth conditions and growth stages. In addition, heavy ion suppression phenomena, which can affect identification of specific proteins, are less problematic when only spectral profiles are being compared. In contrast to Valentine et al. [41], Ruelle et al. [42] observed significant differences in the mass spectra of bacteria when grown on different media.

We have developed methods and specific databases for rapid analysis of bacteria in ballast water, using MTB, for the first time, which can allow the screening of large numbers of bacteria. We further concur with other studies [43], [44] that MTB, when combined with an appropriate marine oriented database, is an extremely useful method when other approaches such as 16S rRNA gene analysis cannot differentiate bacterial isolates. As suggested by Demirev and Fenselau [45], identification of microorganisms within mixtures will become one of the next targets for improvement of the direct characterization of microorganisms (Metaproteomics). There is also a growing need for the standardization of sample preparation and analysis methods for MTB in order to generate reliable, widely accessible libraries for identification of bacteria from specific ecological niches [45], [46]. We therefore suggest a universal method for indicating the minimum reporting requirements for MTB be put in place as already established for proteomics methods [47], [48].
